# Action of Metals on Bacteria

**Published:** 1914-09

**Authors:** 


**Action of Metals on Bacteria.** Pop. Elect. and Mod. Mechanics, August, 1914. Any metal that arrests the growth of bacteria has a distinct bactericide action, this effect extending beyond the actual contact of a disc of metal with the culture. The effect also differs for various bacteria. Pure gold, freshly burnished has no action on bacteria. The same is true of nickel, platinum and a few others. Cadmium, copper, brass (a compound), zinc and silver have distinct bactericide power—a fortunte circumstance when we consider that these metals include the majority used for money.

